# Electron-beam patterned self-assembled monolayers as templates for Cu electrodeposition and lift-off

**DOI:** 10.3762/bjnano.3.11

**Published:** 2012-02-06

**Authors:** Zhe She, Andrea DiFalco, Georg Hähner, Manfred Buck

**Affiliations:** 1 EaStCHEM School of Chemistry, University of St. Andrews, KY16 9ST, U.K; 2 School of Physics and Astronomy, University of St. Andrews, KY16 9ST, U.K

**Keywords:** electrochemical nanotechnology, electrodeposition, lithography, metallic nanostructures, self-assembled monolayers, thiols

## Abstract

Self-assembled monolayers (SAMs) of 4'-methylbiphenyl-4-thiol (MBP0) adsorbed on polycrystalline gold substrates served as templates to control electrochemical deposition of Cu structures from acidic solution, and enabled the subsequent lift-off of the metal structures by attachment to epoxy glue. By exploiting the negative-resist behaviour of MBP0, the SAM was patterned by means of electron-beam lithography. For high deposition contrast a two-step procedure was employed involving a nucleation phase around −0.7 V versus Cu^2+^/Cu and a growth phase at around −0.35 V versus Cu^2+^/Cu. Structures with features down to 100 nm were deposited and transferred with high fidelity. By using substrates with different surface morphologies, AFM measurements revealed that the roughness of the substrate is a crucial factor but not the only one determining the roughness of the copper surface that is exposed after lift-off.

## Introduction

**Figure 1:**
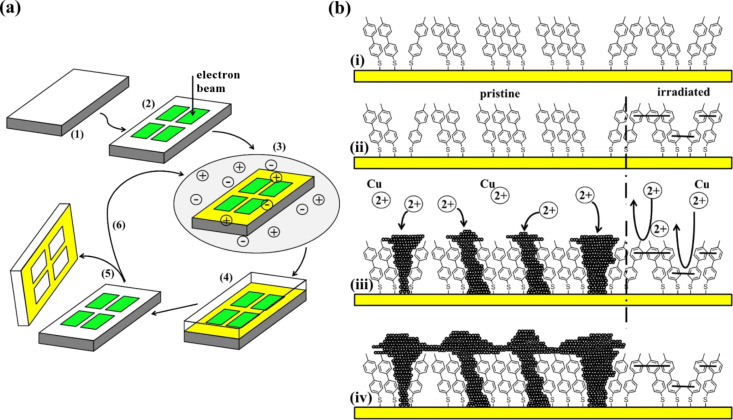
(a) Scheme of SAM controlled electrodeposition and lift-off of metal structures. Starting from a uniform SAM of MBP0 (1), patterning is accomplished by e-beam lithography (2). Acting as a negative resist, electrochemical metal deposition (3) selectively occurs only in the nonirradiated areas. The low surface energy of the CH_3_ terminated SAM enables the transfer of the metal pattern to an insulating substrate (4,5) and reuse of the master pattern (6). (b) Illustration of patterning and deposition processes on the molecular scale. When a pristine aromatic SAM (i) is irradiated by an e-beam, cross-linking of molecules results in bridging of defects (right of ii). The resulting passivation confines the deposition to areas of the native SAM (iii). Metal nucleation starts at the bottom of the SAM and deposits grow in a mushroom-type fashion until they coalesce to form a film (iv).

Covering the range from tens of micrometers down to nanometers, the scope of applications of metal structures in electronics [1–2], sensing [3–7], electrochemical analysis [8], optics and imaging [9–12] will vitally depend on the extent to which the feature size that is required for a particular application can be achieved by processes that enable an affordable high-throughput production. Commonly pursued routes to match resolution with simplicity are based on schemes involving templated deposition on a reusable master substrate followed by a transfer of the structure to the substrate of interest. A key point underlying these processes is to exploit differences in the interfacial forces between the deposited material and the different substrates [10,13–17]. Among the various deposition techniques [18], which also include evaporation [19–20], chemical vapour deposition (CVD) [21–22] and electroless deposition [22–24], electrodeposition [25–28] offers interesting perspectives, in particular at the nanoscale, due to the level of control over the deposition process. The electrochemical approach combines favourably with self-assembled monolayers (SAMs) as it enables the scheme illustrated in Figure 1a [15]. On the one hand, metal can be selectively deposited by using patterned SAMs, which act as template by defining electrochemically active and passive areas of an electrode [25–26,29–33]. On the other hand, the control of interfacial energies afforded by SAMs enables the lift-off and transfer of deposited metal structures. Since a number of techniques exist which cover the range from macroscopic to nanoscopic dimensions [30,34–38] the combination of patterned SAMs and electrochemistry offers a flexible approach for the generation of metal structures.

While structured SAMs exhibiting electrochemical contrast can be made from two different types of molecules that differ in their blocking properties [15], electron-induced modification of a single component SAM is an alternative that is particularly attractive for providing access to the nanoscale, since e-beam lithography as a high-resolution technique can be employed [26,30]. However, the effect is strongly dependent on the type of SAM [25,30,39–40]. Aliphatic SAMs degrade upon exposure to electrons (positive-resist behaviour), in contrast to aromatic SAMs in which the molecular structure of the SAM is essentially preserved [40] apart from the cross-linking of the aromatic moieties. The rather ill-defined electron-induced degradation of aliphatic SAMs makes it very difficult to control electrodeposition and adhesion of a deposit precisely, whereas an aromatic negative-resist SAM does not have this problem. Therefore, for the scheme outlined in Figure 1a, a negative-resist behaviour employing aromatic SAMs is preferred. As illustrated in Figure 1b the effect of electron irradiation is a cross-linking of the aromatic units, which results in the elimination of defects through which metal ions can penetrate the SAM and be reduced at the SAM–substrate interface. In contrast to a scheme that involves complexation of metal ions with the SAM [41–43] and in which the metal is deposited on top of the SAM, the mechanism explored in the present paper relies on defect-mediated deposition, i.e., the metal nucleation takes place at the SAM–substrate interface at sites of structural imperfections in the monolayer. Since the metal deposit grows in a mushroom-type fashion the contact area and, thus, adhesion between the deposited und substrate metal is greatly reduced. The poor adhesion between the metal deposit and the SAM makes the lift-off possible by simple breaking of the stem of the mushrooms [15,17]. Even though it is not the focus of the present paper, we note that if the patterned SAM layer does not deteriorate during the lift-off process it may serve as a master that can be straightforwardly reused [15]. This is of particular advantage for small-scaled structures in which patterning becomes increasingly time-consuming and expensive.

The feasibility of this SAM based deposition and lift-off scheme has been demonstrated for different metals and alloys such as Cu or CoNiFe with uniform SAMs [17,19–20,44–45] and for micrometer-sized Cu structures with a binary SAM consisting of ω-(4'-methylbiphenyl-4-yl)methanethiol (CH_3_–C_6_H_4_–C_6_H_4_–CH_2_–SH, MBP1) as a nonblocking and hexadecane thiol (CH_3_(CH_2_)_15_SH, MC16) as a blocking thiol [15]. The present paper is an investigation of a scheme for creating surface features with smaller dimensions by using e-beam patterning of a single-component SAM of ω-(4'-methylbiphenyl-4-yl)thiol (CH_3_–C_6_H_4_–C_6_H_4_–SH, MBP0). While selective deposition based on e-beam-modified aromatic SAMs has been demonstrated before [25–26,33], with features down to about 50 nm [26], a transfer of the metal structures has not been reported. It is the focus of the present paper to study steps 1–5 of the deposition–lift-off process depicted in Figure 1a, by using an e-beam-patterned SAM, and to investigate the mutually dependent parameters that are crucial for determining key aspects such as the achievable feature size, the precision of the structure, and the fidelity of the lift-off process.

## Results and Discussion

Guided by the scheme depicted in Figure 1, the presentation of the results is organised into two sections discussing electrodeposition and lift-off.

### 1. SAM templated metal deposition

#### General aspects

Analogous to unmodified uniform electrodes [46], we assume that the initial stages of the deposition process can be described by the simple case of a time-independent nucleation rate

[1]
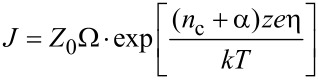


where *Z*_0_ [cm^−2^] is the number density of sites on the substrate where nucleation can occur. Ω is a frequency factor, which, besides other quantities, depends on the concentration of metal ions according to 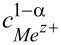
with α as the charge-transfer coefficient; *n*_c_ is the size of the critical nucleus, *e* the electron charge, and η = (*E*^0^ − *E*) is the overpotential (*E*^0^ = standard potential). From Equation 1 it is seen that the nucleation rate increases exponentially with the overpotential. Another point is that a critical overpotential η_crit_ exists, below which the nucleation rate becomes very small. These two points together are very important as they are the key to high-resolution patterning. A double-pulse-polarisation scheme is applied in which an initial nucleation phase at an overpotential that is significantly larger than η_crit_ is followed by further growth at lower overpotentials, resulting in the achievement of high contrast between areas that differ in η_crit_.

**Figure 2:**
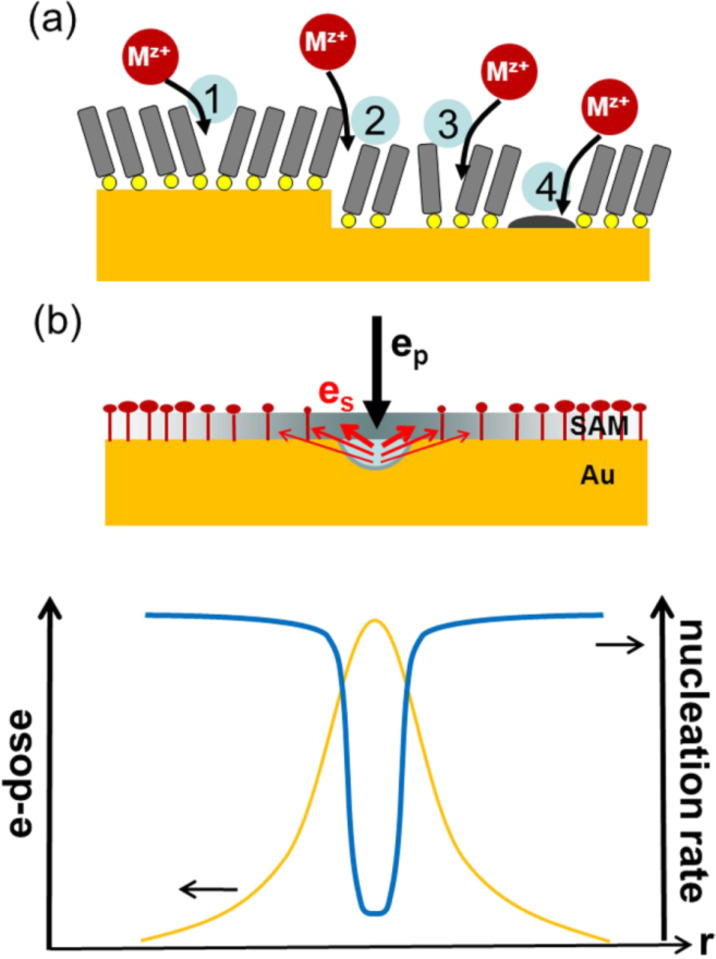
(a) Illustration of different types of defects in a SAM. Domain boundaries (1) and substrate steps (2) as intrinsic defects; missing molecules (3) and contaminations (4) as extrinsic defects. (b) Metal electrodeposition on a negative-resist SAM patterned by e-beam lithography. Top: A primary e-beam, e_p_, generates a spatial profile of secondary electrons, e_s_. The resulting gradient in the cross-linking of the SAM yields a gradient in the density of nucleation sites for the metal mushrooms. Bottom: Illustration of the inverse relationship between irradiation dose and nucleation rate.

For the defect-mediated metal deposition on a SAM modified surface (see Figure 1b), nucleation can occur at different types of defects, as illustrated in Figure 2a and discussed in more detail in [47]. Imperfections such as domain boundaries, substrate steps, missing molecules or contaminations can all serve as nucleation sites. Since reduction of the metal ion is determined by tunnelling of the electron, discharge is much more likely to occur close to the Au substrate than at the outer surface of the SAM. Therefore, nucleation starts preferentially at defects through which the ions can penetrate the layer and, thus, approach the Au surface more closely. The probability that an ion penetrates is dependent on the detailed nature of the defects, and thus the rate at which ions are discharged and at which the critical nucleation size is reached can vary substantially for the different types and sizes of defects. Note that the defects are not necessarily static, i.e., potential-dependent changes or fluctuations in the SAM structure also have to be considered, which makes *Z*_0_ a dynamic quantity. Another factor affecting the nucleation rate is specific to metals that bind more strongly to the thiol head group than the original substrate metal. In this case the metal deposited at defects can easily intercalate and diffuse at the SAM–substrate interface [48].

In the case of templated deposition by means of an e-beam patterned SAM, *Z*_0_ becomes a function of the exposed topology. For aromatic SAMs, such as MBP0, which exhibit negative-resist behaviour, the density of nucleation sites *Z*_0_ is determined by the extent of cross-linking of the molecules. While the exact relationship between the defect size in the SAM and the nucleation probability is not known, a nonlinear behaviour can be expected due to the exponential dependence of the electron transfer on the distance between the ion and the metal surface. Reducing the size of defects by cross-linking should strongly decrease the nucleation probability and, thus, result in a substantial reduction in the nucleation density. This is illustrated in Figure 2b in which a spatial profile in the irradiation dose by e-beam lithography generates an inverted profile in the nucleation rate. It is noted that the cross-linking in the SAM is primarily caused by low-energy electrons (<100 eV) [39–40], and, therefore, the spatial resolution is determined by the distribution of secondary electrons, e_s_, rather than by the one of the high-energy electrons, e_p_, of the primary beam. If a pulsed deposition is used, rather sharp boundaries in the deposition should be possible since two nonlinear effects are superimposed, i.e., the one due to cross-linking and the one due to the overpotential according to Equation 1. The precision at which the contour of a metal deposit can be defined is ultimately dependent on two factors. The first one is the gradient in the nucleation rate; the second one is the density of nucleation sites. Although one seeks to maximise the latter, this is ultimately defined by the defect density in the native SAM, which is thus the limiting factor in the achievable resolution.

#### Experiments

**Figure 3:**
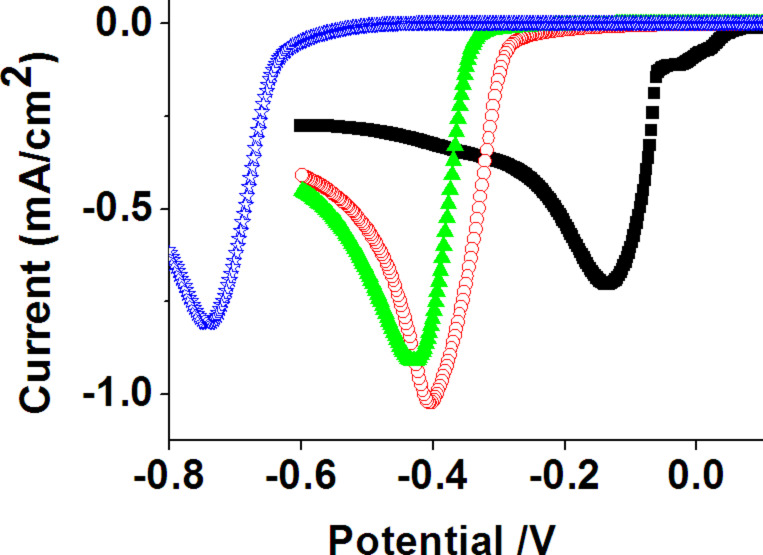
Linear-sweep voltammograms comparing the electrodeposition of Cu on clean (black squares) and SAM modified Au/Si electrodes, from a 50 mM CuSO_4_/0.1 M H_2_SO_4_ electrolyte. SAMs were prepared by 24 h immersion of Au/Si substrates into solutions of octadecane thiol (blue stars) at room temperature and of MBP0 at room temperature (red circles) and 65 °C (green triangles).

**Study of deposition parameters:** Prior to metal deposition on e-beam-patterned MBP0-SAMs, the pristine, uniform monolayers were studied and their passivating properties compared with reference systems previously studied in the literature. As seen from Figure 3, the onset of Cu deposition is shifted to more cathodic potentials for the MBP0 coated electrode compared to the clean Au surface, similar to alkanethiol SAMs [29–30,49–50] and other biphenyl based thiols previously studied [15,25–26,33]. The shift of about −0.27 V to +0.3 V is, however, significantly smaller compared to a long chain alkanethiol such as octadecanethiol for which the shift amounts to about −0.6 V. We note at this point that both the sharpness of the onset of deposition and the value of the peak potential are significantly dependent on the quality of the SAM. An important parameter is the cleanliness of the substrate prior to SAM formation [48]. Contaminations result in pinholes in the SAM (defect 4 in Figure 2a) and as a consequence the cyclic voltammograms (CVs) show an earlier onset of deposition and an initially much more gradual increase than those shown in the CVs of Figure 3. When small structures are desired, the preferential nucleation at such extrinsic defects is unfavourable as they are only present at low density, and it is the nucleation density which ultimately limits the feature size. Another parameter is the preparation temperature, for which a higher temperature, in general, improves the crystallinity of the SAM, i.e., increases the domain size. As seen from Figure 3 this influence is rather small for MBP0 and does not, in fact, lie unambiguously outside the range of sample-to-sample variations, which is in agreement with the overall poor crystallinity of this type of SAM [51]. For this reason samples prepared either at room temperature or elevated temperature were used throughout the experiments.

The selective deposition on a patterned SAM depends on a number of parameters, some of which exert an opposite influence on the deposition. As outlined above, on the one hand, a more negative deposition potential increases the nucleation density and, thus, improves the contour definition of the Cu pattern and the achievable resolution. On the other hand, it reduces the deposition contrast between irradiated and nonirradiated areas since defects in the irradiated SAM are unlikely to be fully eliminated.

**Figure 4:**
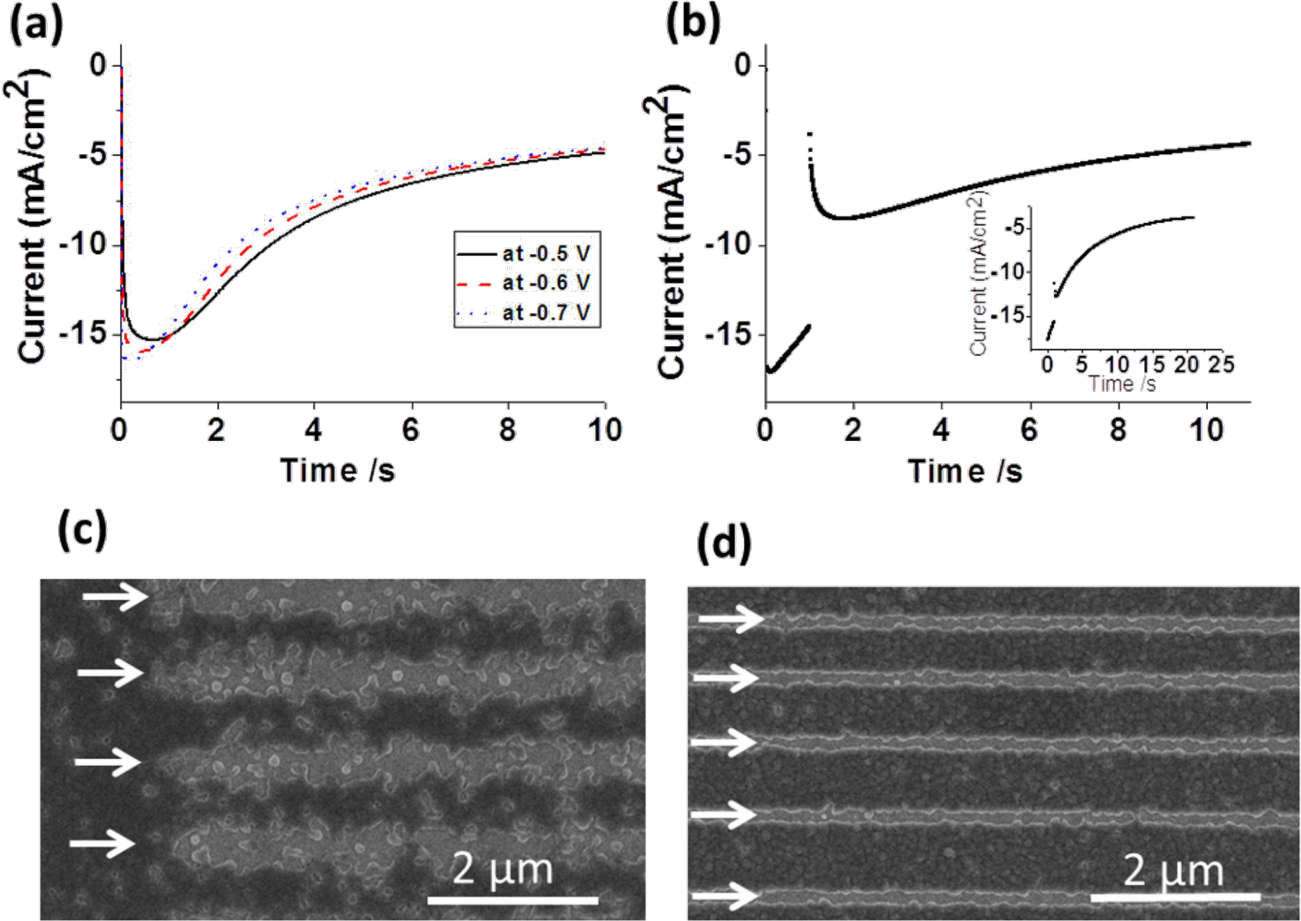
(a,b) Chronoamperograms of single potential (a) and double potential (b) deposition processes on a uniform MBP0-SAM on Au/Si. Potentials of *I*–*t* curves in (a) are −0.5 V (black line), −0.6 V (red dashed line) and −0.7 V (green dotted line) and in (b) −0.7 V for 1 s, −0.35 V for 10 s on MBP0/Au/Si. For comparison an *I*–*t* curve for a clean Au/Si substrate and identical deposition conditions is shown in the inset. (c,d) SEM images of Cu deposition on e-beam-patterned SAMs. Lines indicated by arrows were written with an electron dose of 800 mC/cm^2^ in both cases. Deposition was carried out in (c) at −0.5 V for 15 s, and in (d) at −0.7 V for 1 s and at −0.35 V for 10 s.

For this reason the deposition process was investigated by chronoamperometry. Figure 4a shows *I*–*t* curves of a uniform, pristine MBP0-SAM recorded at three different potentials.

All curves show the characteristic shape of a nucleation-and-growth process. In the initial stage, nucleation is inhibited since Cu reduction is limited by the SAM [45,52]. The current increases due to the formation of nuclei and mushroom structures at defects in the SAM (Figure 1b). At this point the electrode surface can be described by a statistical array of nanoelectrodes. Subsequently the current becomes transport-limited and, therefore, passes a maximum after a given time, which becomes shorter with higher cathodic potential. Diffusion-controlled growth is reflected by a decreasing current whose time dependence evolves into that of a flat electrode upon overlap of the diffusion fields of the mushrooms [53]. This is the region beyond 7 s where the curves adopt an identical shape. An optimisation of the conditions has to take into account three factors: The gradient of the cross-linking, the potential affecting the nucleation density, and the time.

Deposition on an e-beam-patterned MBP0-SAM under the condition of a constant potential is shown in Figure 4c. The SEM image showing Cu free lines about 400 nm wide clearly demonstrates the passivation of the SAM by e-beam-induced cross-linking, which either seals the defects in the SAM or reduces them to a size such that the overpotential required for bulk metal deposition is not reached anymore. It might be worth noting that the absence of bulk Cu deposition does not mean that Cu is not deposited at all. Ions can still penetrate and, analogous to underpotential deposition (UPD), be intercalated at the SAM–Au interface. If the rate of penetration is lower than the diffusion rate at the SAM–substrate interface, mushroom formation is suppressed [48]. While the SEM image demonstrates a clear passivation effect, the contour definition is poor and prohibitive for extension to smaller dimensions. In order to improve the contour definition the nucleation density has to be increased, and an obvious way to do this is to increase the overpotential. However, when going to a larger overpotential, one has to bear in mind that the deposition process is a trade-off between different factors. On the one hand, a more negative potential increases the nucleation density but, on the other hand, will reduce the contrast between native and cross-linked MBP0 areas. Furthermore, with increasing density of mushrooms the lift-off will become more difficult. For these reasons we explored a two-step-deposition procedure as illustrated in Figure 4b. A short nucleation step at potentials more negative than for the one-step sample (Figure 4c) is followed by a growth phase at potentials even more positive than for the single-step procedure.

As evidenced by Figure 4d this results in significantly better pattern definition. Besides the improved contour definition it is obvious that the passivated lines are significantly narrower, despite the fact that identical irradiation conditions were used. The reason for this is the cross-linking profile. Even though the primary e-beam is well focused (~20 nm) the cross-linking is caused by the secondary electrons from the substrate, as illustrated in Figure 2b, thus resulting in line broadening and a gradient orthogonal to the line. With increasingly negative potentials the boundary moves towards the line centre, since nucleation is, as discussed above, a complex process that is nonlinearly dependent on the potential and on SAM defects.

**Figure 5:**
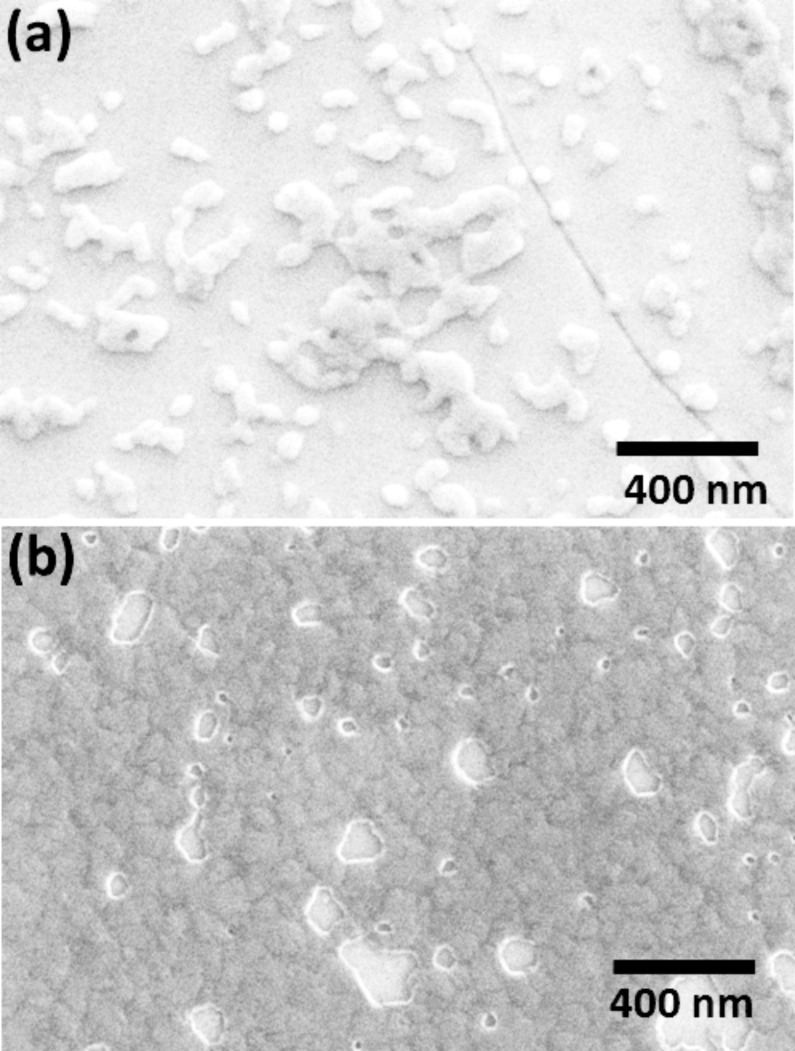
SEM images of Cu nucleation and growth on a MBP0-SAM on Au/Ag/Mica prepared at 65 °C for 24 h. (a) Cu nucleation sites and islands after deposition at −0.7 V for 1 s. (b) Cu layer after nucleation at −0.7 V for 1 s and growth at −0.35 V for 5 s.

The evolution of the deposition for the two-step process is shown in Figure 5, under the conditions depicted in the *I*–*t* diagram of Figure 4b. After 1 s at −0.7 V Cu deposits are observed, which range in size, from small isolated clusters to extended irregularly shaped islands, and demonstrate a significant statistical variation in the nucleation density. After 5 s of further growth at −0.35 V (Figure 5b) a continuous Cu layer is observed with, however, a significant number of holes varying in size, which close upon further deposition.

The statistical variation in the nucleation density evidenced in Figure 5 highlights the limiting factor for the precision of the deposition process, i.e., how sharply the contour between deposition and Cu free areas can be defined. At present the exact relationship between the threshold for nucleation of Cu mushrooms and the nature of the defect is not clear, but the rate at which Cu penetrates through to the Au electrode can be safely assumed to be a decisive factor. Similar to what has been observed for Cu-UPD on a SAM [48], the statistical distribution of rates is determined by the structural quality of the SAM. To improve the precision further one has to develop a process that is independent of the statistical defects in a SAM by, for example, producing a highly passivating SAM and then introduce defects afterwards in a controlled way.

**Figure 6:**
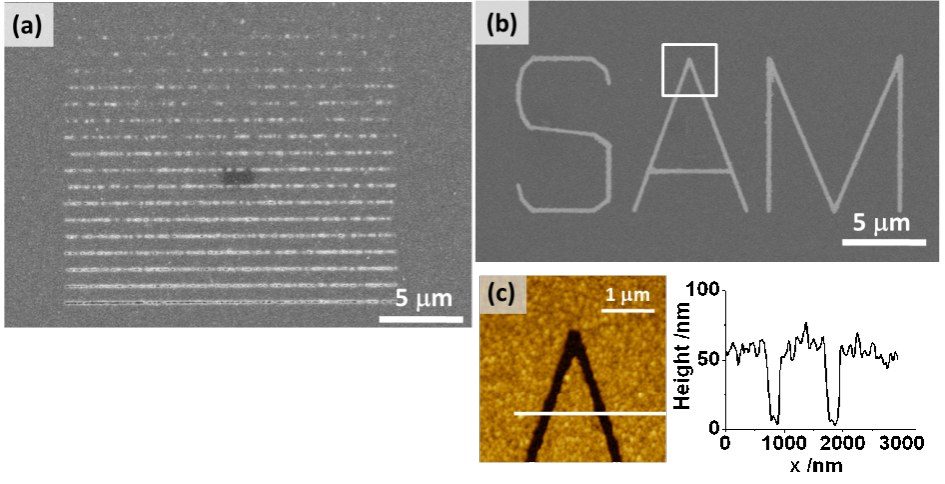
Electrochemical deposition of Cu on e-beam-patterned MBP0-SAM/Au/Si. (a) SEM image of a series of passivating lines written at different doses. The difference in the dose between lines is 25 mC/cm^2^, with 750 mC/cm^2^ as the highest dose for the bottom line. (b) SEM image of “SAM” written with an electron beam at a dose of 1000 mC/cm^2^. (c) AFM image of the area marked by the square in (b) together with a height profile along the line. Deposition conditions: −0.7 V for 1 s, −0.25 V for 20 s (a); −0.7 V for 1 s, −0.35 V for 10 s (b).

**Deposition on e-beam-patterned SAMs:** As discussed above, the extent to which defects in the SAM are modified by electron-induced cross-linking is crucial for the spatial resolution. Therefore, besides the parameters for the electrochemical deposition the influence of the irradiation dose on the quality of the Cu structures was also studied. In a series of lines written by the electron beam, the dose was varied between 50 and 750 mC/cm^2^. As seen from Figure 6a, there is a pronounced improvement in the definition of the lines for which Cu deposition was suppressed. It is noted that the doses needed to produce good contrast in our electrochemical experiment are substantially higher compared to those reported in the literature. For example, for Ni deposited from the gas phase about 45 mC/cm^2^ was used [54]. In electrochemical deposition of Cu on C_6_H_5_–C_6_H_4_SH SAMs [26,33] and CH_3_–C_6_H_4_–C_6_H_4_–(CH_2_)_12_–SH [25] a dose of maximal 80 mC/cm^2^ was used. However, it is difficult to compare the conditions, both with regard to the patterning parameters and the deposition conditions. The yield of the low-energy secondary electrons causing the cross-linking may vary substantially as a primary beam with an energy of 30 keV was used in the present experiments compared to the few hundred eV to 3 keV in the other experiments. Furthermore, the potential in the two potential protocols where nucleation occurs was significantly more negative compared to potentials applied in the one-step-deposition process reported in the literature [26,33]. For the high doses used in this work in combination with the two-step-deposition procedure, an excellent contrast is achieved as demonstrated by the pattern depicted in the SEM image of Figure 6b and the AFM image of Figure 6c showing grooves about 170 nm wide and 60 nm deep.

### 2. Lift-off of Cu structures

With regard to the transfer of the deposited pattern to an insulating substrate we were particularly interested in the following points: (i) The fidelity of the lift-off process; (ii) the morphology of the metal surface originally facing the SAM in comparison with the surface of the growing film exposed to the electrolyte; and (iii) the relationship between the roughness of the substrate and the Cu structure.

**Figure 7:**
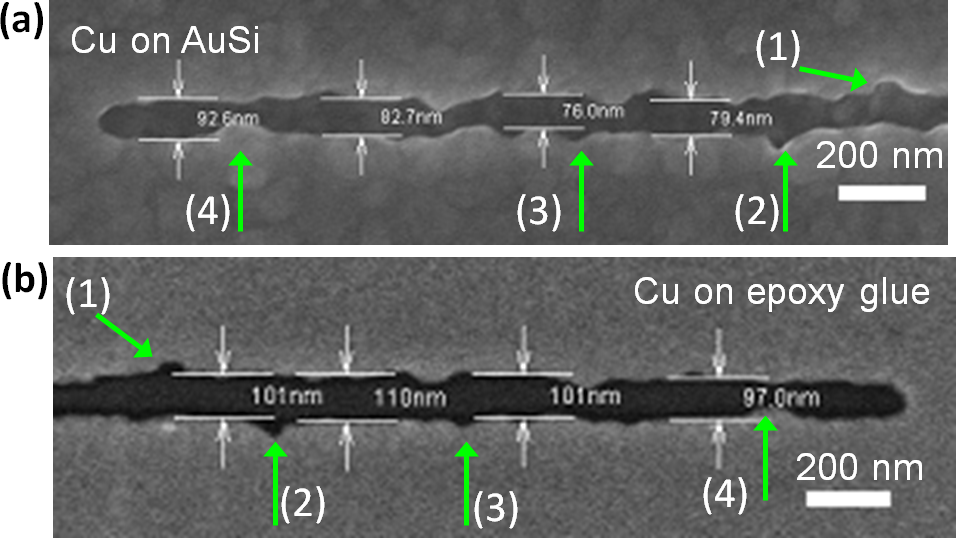
SEM images of a SAM templated copper deposit on the original MBP0 coated Au/Si substrate (a) and after transfer to epoxy glue (b). The passivating line of the cross-linked SAM was written by using an e-beam dose of 750 mC/cm^2^ . Deposition parameters are −0.7 V for 1 s, −0.25 V for 20 s. The numbered green arrows mark the corresponding features in (a) and (b).

Figure 7, showing a copper structure as deposited and after lift-off, demonstrates that the pattern is transferred without distortion. All features of the trench seen on the original structure (Figure 7a) are precisely reproduced in the structure attached to the epoxy glue (Figure 7b), which, due to the lift-off, appears as a mirror image of the original structure. The fidelity with which the pattern is transferred demonstrates that the simple transfer process is suitable for the routine generation of high-resolution metal patterns on insulating substrates even for significantly smaller structures down to ~50 nm, which have been tried. However, even though the transfer process imposes no restrictions on the feature size, at this point we did not systematically pursue the fabrication of features smaller than those shown here, for reasons that are obvious from Figure 7. There are deviations from the straight boundary line separating the copper-free and deposition areas, by up to 20 nm. This is due to a statistical variation in the nucleation density, which is determined by the random defects present in the native SAM and already addressed above. Another point is an increase in the width of the line by about 20% when going from the structure as deposited (Figure 7a) to that after the lift-off (Figure 7b). We ascribe this to nonvertical growth of the trench walls due to transport-limited deposition, similar to subconformal Cu deposition in microelectronics [55–56].

Besides the definition of the lateral dimensions, another point of interest is the surface topography. Reminding ourselves that the SAM and electrolyte-facing surfaces of the Cu deposition layer become the exposed and buried ones, respectively, after transfer, a comparison of their topography is of interest with regard to potential applications in optics, for example, where the smoothness of films is important.

**Figure 8:**
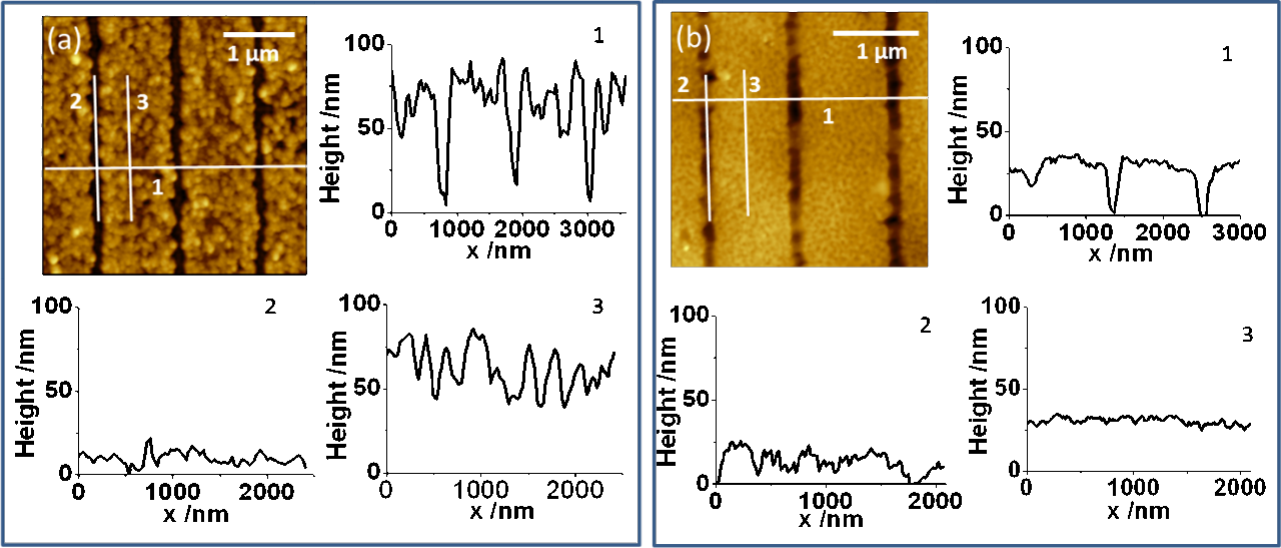
AFM topography images of Cu electrodeposited onto an e-beam-patterned MBP0-SAM on Au/Si (a) before and (b) after lift-off. The height profiles shown are taken along the numbered cross-sections. Lines of cross-linked MBP0 were written by using a dose of 1000 mC/cm^2^. The parameters for Cu deposition were −0.7 V for 1 s, −0.35 V for 10 s.

Figure 8 shows a compilation of AFM images comparing the structure as deposited on a MBP0 patterned Au/Si substrate with the one transferred to the epoxy glue. Parallel lines about 1 μm apart were written into the SAM by e-beam lithography. As inferred from the difference between the grooves, where the cross-linked MBP0-SAM inhibits deposition, and the areas of deposition, the two-step deposition involving a 10 s growth period yields a thickness of the Cu layer of about 70 nm (Figure 8a, curve 1).

Comparison of the height profiles inside and outside of the grooves (Figure 8a, curves 2 and 3) shows that the growing surface of the Cu deposition is significantly rougher than the original substrate. This is very different from the Cu surface facing the SAM which is depicted in Figure 8b. It is seen from the height profile (Figure 8b, curve 3) that this Cu surface has a corrugation comparable to that of the substrate (Figure 8a, curve 2). In contrast, the profile along the line is less-smooth compared to the corrugation in the original groove. Together with the line depth (Figure 8b, curve 1), which is significantly smaller than for the original grooves and ranges between 10–40 nm, this demonstrates that the filling of the grooves with epoxy glue is rather incomplete. Taking into account that the fairly viscous glue is applied under ambient conditions, we consider air trapped in the grooves to be the major reason. Unfortunately, further studies excluding air, in particular to see whether the glue in the lines can be made coplanar with the metal surface, were impossible, since we could not apply the epoxy glue under vacuum.

**Figure 9:**
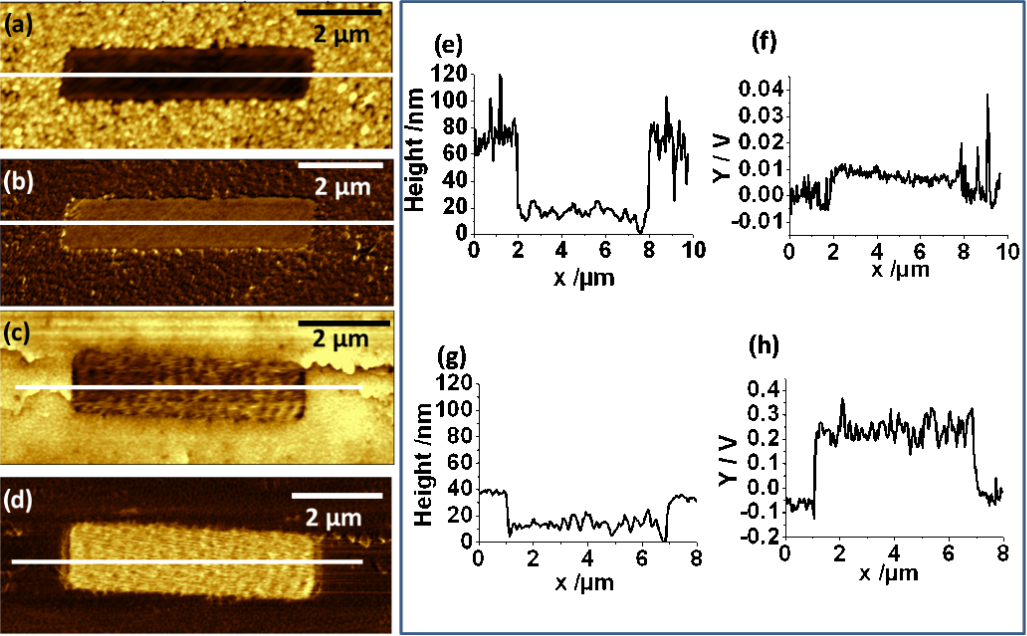
AFM images of Cu electrodeposition onto a MBP0/Au/Si sample demonstrating the quality of passivation of the cross-linked MBP0-SAM. Topography (a,c) and friction (b,d) images of the Cu structure as deposited (a,b) and after transfer to epoxy glue (c,d); (e) height and (f) friction profile along the line for Cu as deposited; (g,h) corresponding profile for the lifted-off structure. The 5 × 1 μm^2^ rectangle of cross-linked MBP0-SAM was generated with an electron beam dose of 500 mC/cm^2^. Conditions for the two-step electrodeposition were −0.7 V for 1s and −0.35 V for 10 s.

Similar results were observed with a wider trench structure. Figure 9 shows a comparison between the two Cu surfaces analogous to the line structure in Figure 8. Again, the deposition contrast is excellent with a complete suppression of deposition also for this extended cross-linked area. The thickness of the Cu deposit was approximately 70 nm as seen from the line profile in Figure 9a, i.e., the same as measured for the line structure (Figure 8) for which the deposition parameters were the same. The depth between the Cu surface and the copper-free area was 30 nm after lift-off, revealing an incomplete filling of the trench by the glue, which is again likely due to trapping of air. On comparison of the friction images of the Cu structure as deposited and after lift-off (Figures 9b and 9d), a very different friction contrast is seen between deposition and Cu free areas according to the mechanical properties of the materials. While in both cases the friction inside the trench is higher than on the Cu deposit, the difference between the two areas is more than 30 times larger between the rather soft epoxy glue and Cu, compared to SAM/Au and Cu.

**Roughness measurements:** Since, as evident from Figures 8 and 9, the Cu surface facing the SAM is substantially smoother than the opposite one with a roughness close to that of the substrate, the influence of the substrate quality was studied in more detail.

For this purpose substrates with different degrees of roughness were compared. Besides Au/Si whose morphology is determined by small crystallites of different orientations, Ag/mica and Au/Ag/mica substrates were used because Au and Ag can be grown epitaxially on mica [57–58], and this results in less corrugated films with a well-defined (111) orientation of the crystallites and much larger terraces. The reason for using Ag either as a substrate directly, or as interlayer, is that Au adheres poorly to mica. While the poor adhesion of Au has been taken advantage of for the generation of ultraflat Au substrates through the template-stripping method [59–60], it is a limiting factor for our scheme. Even though transfer using Au/mica can be achieved to some extent, the parameters must be so narrowly defined as to prohibit a reliable, routinely applicable process. By using silver this problem is significantly alleviated.

**Figure 10:**
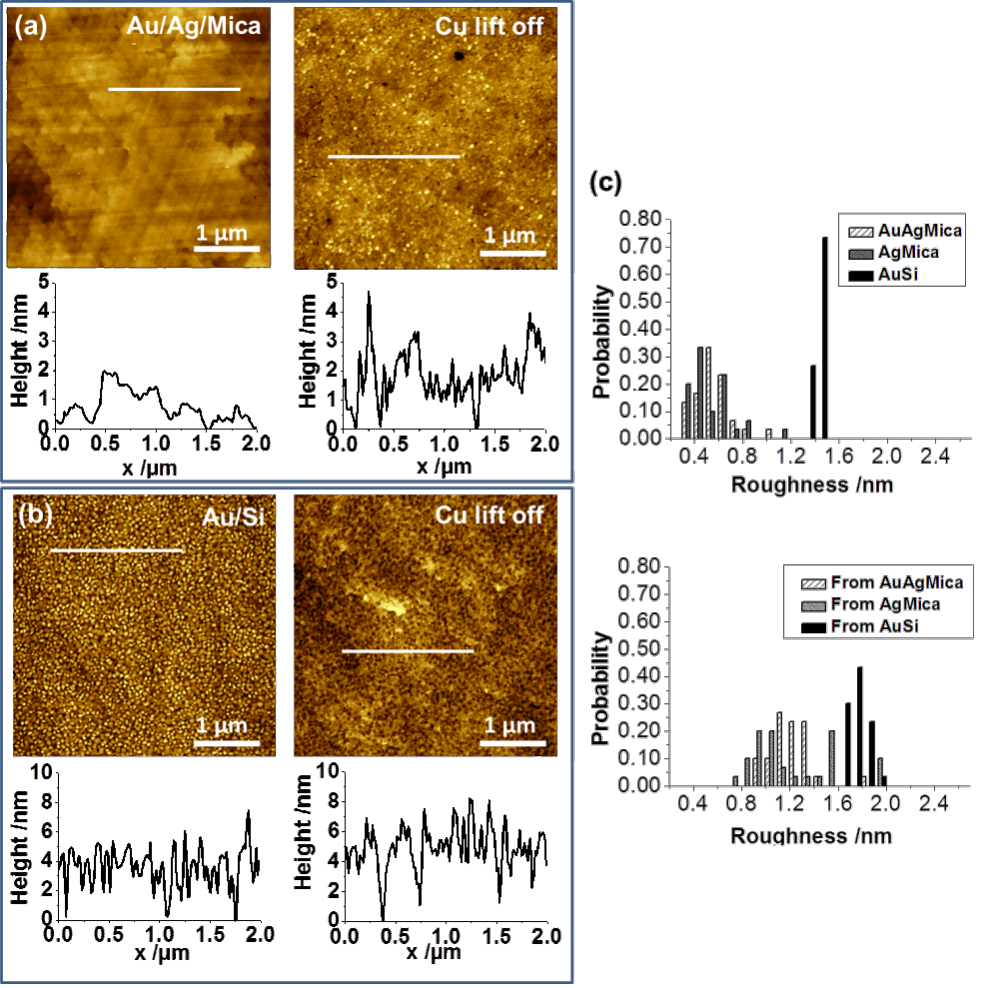
(a,b) AFM topography images and height profiles along the lines indicated, comparing the roughness of different substrates with the corresponding surface of the Cu film after lift-off; (a) Au/Ag/mica (b) Au/Si. Conditions for Cu deposition: −0.8 V for 2 s and −0.35 V. (c) Roughness histograms of substrates (top) and Cu surfaces after lift-off (bottom) from 30 measurements of areas 1 × 1 μm^2^ in size for each surface.

In a series of experiments Cu films were uniformly deposited on MBP0 modified substrates and subsequently transferred to epoxy glue, and the surfaces were then compared with the original substrate. Representative examples for Au/Si and Au/Ag/mica are shown in Figure 10a. The latter is also essentially identical to Ag/mica (not shown) as inferred from the histograms shown in Figure 10b and Table 1, which compiles the averaged root-mean-square (RMS) values and their variations expressed as the standard deviation σ. Figure 10b represents the results from 30 RMS measurements for each substrate and with values grouped into intervals of 0.1 nm.

**Table 1:** Statistical analysis of the roughness measurements for different substrates and corresponding Cu surfaces after lift-off. Root-mean-square (RMS) average determined from 30 measurements of areas 1 × 1 μm^2^ in size. σ is the standard deviation of the RMS values.

substrate		RMS average [nm]	σ [nm]

Au/Si	substrate	1.42	0.03
	Cu lift-off	1.74	0.07
Au/Ag/Mica	substrate	0.57	0.15
	Cu lift-off	1.22	0.17
Ag/Mica	substrate	0.54	0.18
	Cu lift-off	1.22	0.35

From the histograms and the tabulated values one can infer that, on the one hand, the substrate substantially influences the roughness of the Cu surface but, on the other hand, is not the limiting factor. The improvement in the surface roughness of the Cu structure from 1.74 nm to 1.22 nm upon changing from Au/Si to the mica-based substrates is evidence for the former, whereas the increase in roughness of the lift-off Cu structure compared to the substrates reveals the latter and demonstrates that the deposition process is also crucial for the topography. This is not unexpected, as the roughness must be dependent on the morphology of the mushrooms, in particular at the point of coalescence. In this context we note that deposition on Au/Si under slightly different conditions such as −0.7 V for 1 s and −0.25 V for 20 s for nucleation and growth, respectively, can result in a slightly smoother surface of the deposit, hence indicating that the deposit does not exactly reproduce the contour of the SAM surface. While the roughening of the Cu structure by a factor of two compared to the mica substrate is substantial, its cause is not clear at present. The mushroom morphology, as the factor ultimately limiting the flatness, cannot account for it. Even though it is not clear where the stems of the mushroom break during the lift-off process, with a maximal height of about 1.5 nm and a density of less than 1 per 100 nm^2^ the contribution to the roughness must be significantly smaller. There is scope for further improvement, as the optimisation of parameters, such as nucleation potential, time, growth potential, and the use of additives [45], was not systematically investigated. Furthermore, the lift-off process has not been studied in detail and it is currently an open question as to what extent the forces that act during the curing of the epoxy and the lift-off process influence the roughness of the exposed metal surface.

## Conclusion

The possibility to control both electrode activity and interfacial energies by means of a patterned SAM is exploited in a scheme to generate metal structures on an insulating substrate by a simple electrodeposition/lift-off scheme. An important point with regard to the realisation of small features is that the scheme relies on a trade-off between the nucleation density and the control of adhesion. Since the deposition is defect-mediated, an increase in the number of defects will necessarily result in higher adhesion. While this will become a resolution-limiting factor at one point, the currently realised structural features of down to around 50 nm are limited by the precision at which nucleation can be controlled. Rather than relying on statistical defects originating from the SAM preparation itself, the controlled introduction of defects ex post facto into a well passivating SAM should be the way forward towards significantly higher resolution. Considering the excellent blocking of metal deposition by the cross-linked MBP0-SAM, inducing defects by means of a focused ion beam seems like a promising strategy.

An advantage of the scheme is that the metal surface exposed after lift-off is very smooth and, thus, very similar to template-stripped uniform films but with the additional feature of small-scale patterns. Even though the substrate roughness plays a crucial role for the topography of the film, there are still contributions from additional factors that have yet to be elucidated. One obvious point is a further optimisation of the deposition parameters with regard to the mutual interplay between growth rate and morphology. So far the scheme has been demonstrated for Cu, and it will be of interest to extend this to other metals, such as Ag or Au, and to see how the different interactions between these metals and the SAM will affect the deposition process. Another aspect is to explore the repeatability of the process, i.e., the stability of the SAM patterns upon multiple cycling comprising all of the steps 2–6 as depicted in Figure 1a.

## Experimental

**Substrates, SAM preparation and patterning:** Two types of gold substrates purchased from Georg Albert PVD, Germany were used: (i) 100 nm of Au evaporated onto a Si(100) wafer with a 5 nm titanium interlayer; (ii) 300 nm of Au on 300 nm of silver on mica slides. Both Ti and Ag served as adhesion promoters. Substrates were cut into 3–5 cm^2^ pieces. SAMs were prepared by immersion of the substrate into a 100 µM solution of ω-(4'-methylbiphenyl-4-yl)thiol [51] (CH_3_–C_6_H_4_–C_6_H_4_–SH, MBP0) in ethanol, either at room temperature or at 65 °C, for 24 h. Samples were then rinsed with ethanol and blown dry in a stream of nitrogen.

Patterning of the SAM was performed by e-beam lithography (RAITH Elphy Plus/LEO 1530 hybrid system) with a 30 kV beam, and exposures varied between 40 and 1000 mC/cm^2^. Patterned SAMs were reimmersed in MBP0 solution at room temperature for 8 h.

**Electrochemistry:** Using an Eco Chemie AUTOLAB PGSTAT128N and NOVA 1.4 software, the electrochemical experiments were performed in a home-built cell with a standard three-electrode configuration. Cu wires served as both reference and counter electrodes. The area of the working electrode was 40 mm^2^. Electrodeposition of Cu was carried out with a 50 mM CuSO_4_/H_2_SO_4_ solution of about pH 1 (chemicals from Sigma-Aldrich, 99.999%). After electrochemical deposition the substrates were rinsed with deionised water and dried under a stream of nitrogen.

A two-potential deposition was employed for deposition of the Cu structures. Typical values for the two steps were in the ranges between −0.6 and −0.8 V for 1–2 s and between −0.25 and −0.35 V for 10–20 s, respectively.

**Lift-off of electrodeposited Cu:** Both uniform films and patterns were lifted off mechanically by applying epoxy glue (Araldite rapid set), which was cast by placing a Teflon plate with a hole of 6 mm diameter onto the substrate. Curing of the epoxy was performed at room temperature, typically overnight.

**Characterization:** Cu structures were characterised by scanning electron microscopy (Hitachi S4800) and atomic force microscopy (PicoPlus, Molecular Imaging). Using Veeco NPS10 nonconductive silicon nitride tips (spring constant 0.06 N/m) AFM images were recorded in contact mode by using forces between 7 and 13 nN and scan rates of 0.9 to 1.2 Hz. Images were analysed using either the Picoscan software (Molecular Imaging) or Gwyddion. For the roughness analysis of the AFM topography images (4 × 4 µm^2^, 512 × 512 pixels) images were line-corrected by matching to the height median, and horizontal scars were removed. The root-mean-square values were measured by performing Gwyddion statistical analysis of areas 1 × 1 µm^2^ in size from 4 × 4 µm^2^ sized images (512 × 512 pixels).
